# Dr. Nansen's Message

**Published:** 1889-09-28

**Authors:** 


### The Hospital

A Weekly Institutional Journal of

**Science, Medicine, Nursing, and Philanthropy.**

*For Hospitals, Asylums, Medical Practitioners, Students, Nurses, Families, Parishes, Congregations, and the Charitable Public.*

Vol. VI. No. 157.] [September 28, 18[illegible]

### Dr. Nansen's Message.

Dr. Nansen, who drew a large crowd, especially of ladies, at the British Association last week, reported of the natives he had seen on the West Coast of Greenland that "as they had not been spoiled by the vices of civilisation they were happy, and, though poor, they knew nothing of the miseries of poverty." Happiness is, of course, a relative term, as also are misery and poverty. Dr. Nansen is a young man of splendid physique and brimming energy, who had not time for more than a very hasty glance at the West Coast Greenlanders. His statements must therefore be taken with a due admixture of sober consideration. But it is entirely in accordance with general experience that scattered populations which are compelled, by their environment of climate and soil, to work hard for a livelihood, are more robust in health and ordinarily much happier than the poorer inhabitants of large cities.

Every man and woman in Great Britain who has sense enough to see and understand the terrible straits of poverty, and the consequent abysses of misery into which tens of thousands of the working population of large towns are thereby plunged, will be willing to give at least an occasional five minutes to the consideration of the subject. If it be true that there is not a living man who is not either a Platonist or an Aristotelian, it is equally true that the whole human family are divided into Optimists and Pessimists. Accordingly as a man belongs to one or other of these natural divisions will he be disposed or otherwise to give time and hard thought to the sad problems presented by modern civilisation. That it is the duty of every man, whether optimist or pessimist, to make the most strenuous efforts of which he is capable for their solution, we, at any rate, cannot for a moment doubt.

Dr. Nansen gave his audience a kind of flying message to the effect that the West Coast Greenlanders are happy, though uncivilised and poor; the Rev. Herbert Mills, addressing the same audience on his favourite subject of "Home Colonisation," steadily, and even solemnly, assured them that large masses in this country are steeped in poverty and drowned in misery, although civilisation surrounds them on all sides like a besieging army. The two sets of facts demand to be looked at side by side. It is necessary to repeat, and to insist, that they are facts, concrete and living facts of the present moment, and not mere signs on paper like the opposite sides of an algebraic equation. It is necessary, because the majority of men who read of facts on paper argue them out also on paper, solve the difficulties connected with them on paper, and never think of dealing with them in any fashion at all elsewhere than on paper. To them ten thousand hungry children in Whitechapel, or five thousand fathers and mothers who throw themselves on the poor rates in their desperation, are of no more significance than five thousand potatoes in a market sack, or ten thousand tadpoles in a filthy pond.

But what is the practical use of considering such threadbare and heartbreaking subjects side by side? asks the pessimist. There is no use at all if a man or a community be so sunk in self-indulgence that intellectual effort is distasteful, and practical putting of the hands to work impossible. By all means let the pessimist suck his juiceless orange, and get out of it what satisfaction he can. But upon the hopeful and the resolute an inner and resistless compulsion is laid; they cannot but take such questions in hand, and deal with them as practically and as successfully as they may.

The problem is something like this: If scattered country populations are poor but comparatively happy, whilst large masses in towns are still poorer, and at the same time superlatively miserable, can any means be devised whereby the miserable masses of the poor in towns may at least be put into possession of the comparative happiness of the poor in country districts? Before going any further let every reader ask himself two questions: First, is it desirable, if it be possible, to remove the poorest of the poor from towns to the comparative health and happiness of thinly-populated country districts? And, secondly, if it be desirable, is it possible? Many people will admit that it is desirable, but not possible; many more will not care whether it is desirable and possible or not. For our part we are convinced, and we speak from personal knowledge and experience, that it is both desirable and possible.

In demonstrating the possibility of supplying the miserable poor of large towns with both space and work in the country we need not go further away from London than the county of Kent. Kent has a large agricultural population; but it is quite certain that Kent could find profitable occupation for at least double the number of agricultural labourers of all kinds that it now employs. There are whole districts of that county which for want of skill and capital on the part of the tenant farmer do not produce one-third of the value in crops which the soil is naturally capable of producing. Whilst the hedgerows at this season of the year are thick with wild fruits of all kinds, and every bank and uncultivated corner is a wilderness of rank grass and weeds; whilst the landscape is beautiful with oak and ash and other forest trees, and luxuriant shrubs abound on every side, the crops of wheat, barley, and oats which have just been gathered in from the fields were poorer and more feeble in growth than those that are produced on the stoniest soils and in the coldest districts of the northern highlands of Scotland. Of root crops there are hardly any at all. What are the consequences? The farmer is desperate, the labourer starves, and the small landlord cannot afford so much as a horse to ride on, or even, in some cases, to live in his own house. What is true of Kent is generally true, though, of course, the local circumstances differ somewhat, of almost every other county in England. Far more than twice the number of labourers could be employed on the soil with the greatest possible advantage to farmers, labourers, landlords, clergymen, doctors, tradesmen, and the whole English community.

But how is this to be done, asks the bewildered town dweller, who knows no more of the real life of the country and of its possibilities than he knows of heaven, or of Robinson Crusoe's island? It could be done by the richer landlords if they had the patriotism and the intellectual capacity. That, perhaps, is not to be expected. Wealth and pride are often as destructive of intellectual capacity and of patriotism as they are of other virtues. But there is at least an opportunity before us of making an experiment, and that on a scale sufficiently large to show how it will really work. The Rev. Herbert Mills has got his scheme of "home colonisation" so far advanced as to have made it a subject of discussion by the British Association. Whether anything practical will come of that it is impossible to say. But Mr. Mills and his coadjutors are prepared, as an experiment, to remove bodily from the slums of London or elsewhere five hundred persons or more to some quiet country spot, and there to employ everyone of them either directly on the soil, or indirectly in providing food, clothing, and other necessaries for those who do work on the soil. They will, in fact, plant a colony in an English country district, and make it do all it needs for itself. They are confident that in a few years the colony will be both self-supporting and prosperous. They back up this contention by the undoubted fact that several colonies of the kind are now in existence in Germany and are prosperous beyond all anticipation. If poor German town dwellers can colonise prosperously in this way, all experience assures us that poor English town dwellers can do it better still.

Is the experiment worth making? Every thoughtful or even thoughtless man will admit that it is. Why, then, is it not made? Because Mr. Mills is only a clergyman, and cannot afford to put down twenty-five thousand pounds. Had he been a public singer like Patti, or even a popular actress like Mrs. Langtry, he might have devoted a year's savings to the work, and so have got it done. But a highly-civilised public like our own, which can give its favourite actors and singers twenty or thirty thousand a year, with effusive prodigality, has not given Mr. Mills and his trusted coadjutors a tenth of that money for his home colonisation scheme, although they have been arguing, and convincing, and spending time, and strength, and means on that project for three or four years past. Truly it is a wonderful world! We address, perhaps, from twenty to thirty thousand readers weekly. Is there one among them who, if he had the will, could, by persistent effort, bring Mr. Mills's scheme to a practical experiment within a year of the present time? If there is such a man, or woman, he has offered to him the opportunity of initiating a movement which a Lincoln might have envied, and which even a Moses would hardly have despised.

